# Relationship between Neck Circumference and Non-Alcoholic Fatty Liver Disease in Childhood Obesity

**DOI:** 10.4274/jcrpe.2313

**Published:** 2016-03-01

**Authors:** Nihal Hatipoğlu, Serap Doğan, M. Mümtaz Mazıcıoğlu, Selim Kurtoğlu

**Affiliations:** 1 Erciyes University Faculty of Medicine, Department of Pediatric Endocrinology, Kayseri, Turkey; 2 Erciyes University Faculty of Medicine, Department of Radiology, Kayseri, Turkey; 3 Erciyes University Faculty of Medicine, Department of Family Medicine, Kayseri, Turkey

**Keywords:** Non-alcoholic fatty liver disease, obesity, metabolic values, anthropometric measurements

## Abstract

**Objective:**

The aim of this study was to establish the association between anthropometric parameters and non-alcoholic fatty liver disease (NAFLD) and to determine the most reliable measurement as a parameter in predicting NAFLD.

**Methods:**

Two-hundred fifty-three obese children of ages 10 to 18 years were enrolled in this study. Anthropometric data and metabolic parameters such as fasting blood glucose, insulin and lipid levels, were measured. Liver function tests were assessed. NAFLD was determined by ultrasound.

**Results:**

Most metabolic parameters and anthropometric indices were significantly higher in children with NAFLD. A univariate logistic regression analysis was performed, taking NAFLD status as the dependent variable and anthropometric parameters as the independent variables. NAFLD was affected significantly by the anthropometric values. The multiple logistic regression analysis showed that neck circumference (NC) was the only parameter which determined the risk in both genders. Each 1 cm increase in the NC increased the risk of NAFLD 1.544-fold (p<0.001, 95% confidence interval (CI): 1.357-2.214) in the boys and 1.733-fold (p=0.001, 95% CI: 1.185-2.012) in the girls. Receiver operating characteristic analysis was performed to compare the reliability of anthropometric measurements. NC was observed to be a better indicator.

**Conclusion:**

Measurement of the NC was shown to be associated with NAFLD in children. We suggest the use of NC as a novel, simple, practical, and reliable anthropometric index in predicting children at risk for NAFLD.

**WHAT IS ALREADY KNOWN ON THIS TOPIC?**

In obesity, central body fat is strongly linked to risk of non-alcoholic fatty liver disease (NAFLD) and metabolic complications rather than total body fat. Anthropometric measurements such as body mass index, waist circumference, mid-upper arm circumference providing information about body fat and fat distribution can be used to predict the risk of NAFLD in obese children.

**WHAT THIS STUDY ADDS?**

Besides other anthropometric measurements, neck circumference was significantly related to upper body fat and NAFLD. Neck circumference may be used as an additional useful screening being an inexpensive, practical and reliable anthropometric measure to assess NAFLD in obese children.

## INTRODUCTION

One of the complications of obesity is non-alcoholic fatty liver disease (NAFLD). As in adults, NAFLD has become the most common cause of chronic liver disease in childhood (1,2). Additionally, NAFLD is closely related with insulin resistance, type 2 diabetes mellitus, dyslipidemia, hypertension, metabolic syndrome, and severe cardiovascular complications (3). In obesity, central body fat, rather than total body fat, is strongly linked to risk of NAFLD and metabolic complications (4,5).

Various anthropometric parameters have been developed to determine total body fat and central body fat accumulation. Body mass index (BMI) is used as major index in the evaluation of obesity. Waist circumference (WC), mid-upper arm circumference (MUAC), and waist-height ratio (WHR) are recommended in determining central body fat (6,7,8,9). Recently, a few studies have been reported suggesting that upper body fat accumulation and visceral fat may contribute to the development of risk factors for metabolic disease (5). Neck circumference (NC) has been suggested as a useful tool to determine the upper body fat accumulation (10).

Based on this information, anthropometric measurements providing information about body fat and fat distribution can possibly be used to predict the risk of NAFLD in obese children at a young age. Thus, it would be possible to prevent fatty liver disease in its early stages.

The aims of this study were to determine the relationship between NAFLD and metabolic disorders and to show the reliability of anthropometric measurements including BMI, WC, MUAC, NC, and WHR in detecting cases with NAFLD. We also aimed to find the most reliable and practical measurement among these anthropometric criteria.

## METHODS

A total of 248 children (114 boys and 134 girls between the ages of 6 and 18 years) admitted to our endocrine outpatient clinic because of obesity were enrolled. All children who participated in the study had BMI levels above the 95th percentile according to our reference values (11). The present study was approved by the local ethics committee. Signed consent was obtained from all parents of the children participating in the study. Patients with diseases which may cause obesity such as hypothyroidism, Cushing’s syndrome, those with diseases/deformity affecting anthropometric measurements, patients with hepatitis (viral, congenital) or a history of alcohol use, and children who were using any kind of medicine were excluded. None of the participants had a previous diagnosis of type 2 diabetes or NAFLD.

Chronological age was calculated as the decimal age by subtracting the observation date from the birth date. All anthropometric measurements were performed by the same endocrinologist. Weight, height, WC, NC, and MUAC were measured twice, and the averages were recorded for reference charts. Weights were measured with subjects in minimal (without shoes and with light clothing) underclothes, using a standard beam balance sensitive to 0.1 kg. Heights were determined to the nearest 1 mm using a portable Seca stadiometer.

Body mass index was calculated by dividing weight to the square of height (kg/m2). WHR was calculated by waist circumference divided by height. WC and MUAC were measured as previously described in detail (12). NC was measured using a non-stretch plastic tape measure while the child’s head was being held erect, with the eyes facing forward, and the neck in a horizontal plane at the level of the most prominent portion, the thyroid cartilage. All measurements were taken with the subjects standing upright, with the face directed forward, and shoulders relaxed (8). Systolic blood pressure (SBP) and diastolic blood pressure (DBP) were measured twice in a sitting position after 20 min of rest and the average measurement was recorded.

The Tanner staging was used in the evaluation of pubertal development. However, since the number of our pubertal subjects was limited, subjects at pubertal stages 3 and 4 were combined in the analysis (13).

Blood samples were collected after a 10-hour overnight fast for determination of fasting blood glucose (FBG), insulin, total cholesterol, low-density lipoprotein (LDL), high-density lipoprotein (HDL), and triglyceride (TG) levels as metabolic function tests and alanine aminotransferase (ALT), aspartate aminotransferase (AST), and gamma-glutamyl transpeptidase (GGT) as liver function tests. Biochemical parameters were determined by using enzymatic kits from Roche Diagnostics with a Cobas Integra 800 autoanalyzer. Insulin was measured by the electrochemiluminescence immunoassay method using Roche kits (Roche Diagnostics, Mannheim, Germany).

Homeostasis model assessment for insulin resistance (HOMA-IR) was calculated using the equation: HOMA-IR=Fasting insulin (μU/mL)xfasting glucose (mg/dL)/405 (14).

The ultrasonographic (USG) examinations of all the children were performed using a 3.5 MHz convex transducer (Xario TOSHIBA). All children were evaluated in supine position by the same radiologist. The echogenicity of the liver parenchyma was compared with the right kidney parenchymal echogenicity. USG evidence of NAFLD was based on the bright hepatic echo pattern, increased echo attenuation, and loss of intrahepatic architecture (15).

### Statistical Analysis

The Student’s t-test was used to compare the findings in subjects with or without NAFLD. All statistical analyses were adjusted for pubertal stage and chronological age.

The relationship between anthropometric parameters (BMI, WC, NC, MUAC, and WHR) and metabolic parameters (FBG, insulin, TG, and HDL-cholesterol levels, HOMA-IR, and liver function tests) were evaluated by parietal Spearman correlation test adjusted for age and pubertal stage. Univariate and multivariate logistic regression analysis was performed in which NAFLD was dependent and anthropometric parameters were independent variables in each gender. The univariate and multivariate models were also adjusted for age and pubertal stages. Independent variables without significant effect on NAFLD were eliminated by utilizing the backward stepwise elimination (p>0.1).

In order to test the reliability of anthropometric data to diagnosed NAFLD, receiver operating curves (ROC) analysis was made for each pubertal stage.

## RESULTS

The frequency of a fatty liver in USG examinations was 35.5%. The subjects with and without fatty liver in the two sexes were evaluated separately. In the boys, significant differences in BMI, WC, NC, MUAC and WHR measurements, DBP, insulin, liver function tests, and HOMA-IR were found between subjects with and without NAFLD. HDL levels were lower in patients with NAFLD. In girls, all anthropometric parameters and biochemical values except GGT, total cholesterol and TG were higher and HDL levels were lower in patients with NAFLD as compared to those without NAFLD (Table 1).

Adjusting for age and pubertal stage, the correlation analysis between the anthropometric measurements and metabolic risk factors/liver function tests were performed by gender. According to the results of this analysis, of all anthropometric measurements, positive correlations were detected only between NC and liver function tests (ALT, AST, GTT) in boys. This relationship was not found in the girls. The results of correlation analysis between anthropometric measurements and metabolic parameters are given in Table 2.

Regression analysis was performed to determine the relationship between NAFLD and anthropometric measurements. A univariate logistic regression analysis was performed taking NAFLD status as the dependent variable and BMI, WC, NC, and MUAC as the independent variables adjusted for age and pubertal stages (Table 3). In both boys and girls, NAFLD status was affected significantly by the anthropometric values. After adjusting for age and pubertal stages, the multiple logistic regression analysis and the backward elimination method showed that only NC determined the risk in both genders. Each 1 unit increase in the NC increased the risk of NAFLD 1.551-fold and 1.846-fold (p<0.001, B: 0.613, 95% confidence interval (CI): 1.385-2.462) (p<0.001, B: 0.439, 95% CI: 1.284-1.875) in boys and girls, respectively.

**Figure 1:**
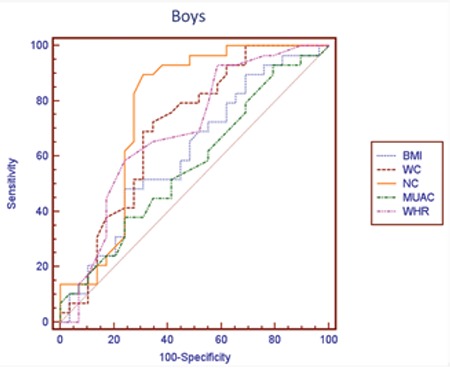
Receiver operating characteristic curves of anthropometric measurements in defining non-alcoholic fatty liver disease in boys. NC: neck circumference, BMI: body mass index, WC: waist circumference, MUAC: mid-upper arm circumference, WHR: waist-height ratio

**Figure 2:**
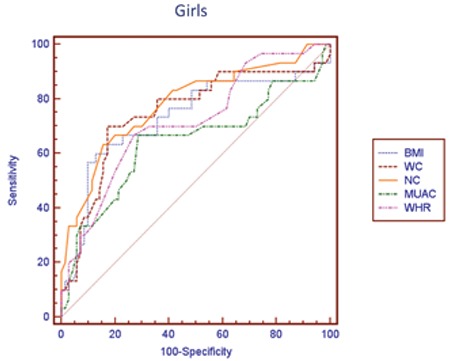
Receiver operating characteristic curves of anthropometric measurements in defining non-alcoholic fatty liver disease in girls. NC: neck circumference, BMI: body mass index, WC: waist circumference, MUAC: mid-upper arm circumference, WHR: waist-height ratio

Similarly, aiming to compare reliability of BMI, WC, NC, WHR, and MUAC measurements for determining NAFLD risk, ROC analysis was performed separately by pubertal stages. While it is possible to use all anthropometric measurements for the assessment of risk in all of pubertal stages, area under curve (AUC) for the NC was higher than the others, except for Tanner stage 3-4 (Table 4, Figure 1, 2). In this pubertal stage, AUC for the WC was found to be higher than the other criteria and similar to NC. Table 5 shown NC cut-off values for determining NAFLD according to pubertal stages.

## DISCUSSION

In the present study, the relationships between NAFLD and metabolic and anthropometric measurements were evaluated. Most of the metabolic parameters and measurements including BMI, WC, NC, MUAC, and WHR were found to be significantly higher in children with fatty liver. In further analysis, we found that NC is the most discriminative measurement that can predict the development of NAFLD.

The global epidemic of childhood obesity has become a serious public health problem and recent studies show that the prevalence of NAFLD in obese children increased (16,17,18,19,20). While the incidence of NAFLD in the general population is 2.6%, this rate increased to 53% in obese children (21). Additionally, a correlation between the degree of obesity and hepatic steatosis has been reported (22). The definition of NAFLD includes a spectrum from simple fatty liver disease to steatohepatitis which is potentially fatal (23).

Even in children with steatohepatitis, NAFLD may still be asymptomatic and is often detected incidentally. Although confirmation of diagnosis can be established by imaging techniques such as computed tomography (CT), magnetic resonance imaging (MRI), MRI spectroscopy, and USG and by increased liver enzyme levels, liver biopsy continues to be the gold standard in the diagnosis of NAFLD (24,25,26). However, liver biopsy is an invasive diagnostic method and it may cause serious complications such as peritoneal hemorrhage (27). Abdominal USG is a safe, non-invasive, and non-expensive diagnostic tool and is applied by most clinicians as the most practical and widely used technique (28,29). In this study, we used USG to identify hepatic steatosis.

Metabolic complications are much more common when body fat is accumulated in the upper body. BMI is an indicator of total body fat, whereas other measurement such as WC, NC, WHR, and MUAC are indicators of body fat accumulation in central and upper body (4,5,6,7,8,9). The relationships of NAFLD with measurements of BMI, WC, WHR, and MUAC have been reported in several publications (4,30,31,32,33,34).

Although both BMI and WC are predictors of NAFLD severity, indicators of central obesity such as WC and WHR are proposed as independent predictors for steatosis (32,33,34). In another study which aimed to determine the relationship between body fat distribution and steatosis, a positive correlation was found between trunk body fat and NAFLD and a negative correlation between thigh fat and liver enzymes (35). In a study on 2111 patients, Ishibashi et al (36) reported that WC shows a positive correlation with visceral adiposity in both genders and that WC may be used as an indicator of fatty liver in males. In a study conducted on Korean adults, it was shown that WHR is as useful as WC to determine NAFLD and as useful as dual x-ray absorptiometry and CT in diagnosis (37).

Compared to reports on adult subjects, there are relatively limited publications about the relationship between NAFLD and anthropometric measurements in childhood. In 69 children with non-alcoholic steatohepatitis, BMI was proposed as a predictor of hepatic fibrosis (38). Oliveira et al (39) showed that each 5 cm increase in WC or a one unit increase in BMI Z-score increases ALT 1.3-fold. Similarly, Lin et al (16) found that the odds ratio of diagnosing NAFLD with USG increased 1.391 times for each 5 cm increase of WC. In a study conducted by el-Karaksy et al (40), a relationship between BMI, subscapular thickness, hip circumference, and WHR, metabolic parameters, and hepatic steatosis is reported in 2-15 years old children. As in adult studies, a relationship between WC and NAFLD has been shown in childhood (30,41).

Neck circumference is accepted as an alternative measurement to detect fat accumulation in the upper body, a finding which is considered to be indicative of a significant metabolic risk factor for type 2 diabetes mellitus and hyperlipidemia in adults (42,43,44). On the other hand, studies about the significance of NC in childhood are relatively new. Recently, both age- and gender-specific NC reference and cut-off values were published in which cardio-metabolic risks related with NC were mentioned (8,45,46,47,48).

This is the first study indicating that there may be a relationship between NAFLD and NC as an indicator of accumulation of fat in the upper body. The only study that can be considered to bear similarity to our rationale showed that dorsocervical lipohypertrophy is related with NAFLD. Dorsocervical lipohypertrophy is also the most reliable measure to estimate the severity of liver inflammation resulting from fatty liver (49).

In our study, we found that NC is correlated with parameters of metabolic risk for the development of NAFLD and also with elevated liver enzymes in males. The results of univariate logistic regression analysis showed that with the exception of MUAC, all parameters were significant to determine NAFLD. In multivariable logistic regression analysis that is independent of puberty and age, we also detected that NC is the most reliable measure to assess fatty liver. The one unit increase in NC has the odds ratio of 1.846 in males and 1.551 in females for NAFLD. In ROC analysis, we found that among other anthropometric parameters and indices, NC is the most reliable parameter indicating the presence of NAFLD except for the midpubertal stage (Tanner stages 3-4). We consider this finding to be related to a change in body fat distribution occurring in this pubertal stage, or to the low sample size. Due to the small size of the sample, we were not able to assess the anthropometric measurements separately in the boys and the girls. In our previous study, the NC cut-off value as an indicator for metabolic syndrome was calculated as 36 cm in boys and 35 cm in girls. In this present study, NC cut-off values to determine NAFLD were calculated according to pubertal stage. The ranges of NC cut-off values were 33 cm for Tanner stage 1 and 36.5 for Tanner stage 5 for both genders.

Body mass index, WC, MUAC, and WHR have been commonly used as indices to determine metabolic risk factors. However, all of these parameters may vary from one person to another. Also, the percentile curves need to be used in the evaluation. NC appears to be a reliable alternative anthropometric parameter to be applied in the assessment of metabolic risk situations. NC, which is an easily measured and practical anthropometric index, may be used to assess upper body fat, and especially for screening NAFLD. The differences between intra-and inter-individual measurements are lower in NC than the other parameters, thus, NC appears to be a reliable and accurate index. In addition, there is no need to take off clothes during the NC measurement (50).

The relatively low sensitivity and specificity of USG analysis to show steatosis or its low capacity to discriminate between hepatitis and steatohepatitis may be considered as a limitation of this study. The fact that anthropometric measurements are indirect measures rather than direct indicators of metabolic risk situations can be listed as another limitation of the study. Finally, our inability to make a gender- and pubertal stage-specific evaluation in ROC analysis because of the smallness of the sample can be considered another limitation of the study.

The present study indicates that NC was significantly related to upper body fat and NAFLD. Since NC is an inexpensive, practical, and reliable anthropometric measurement, we recommend that it can be used as an additional useful screening method to assess NAFLD in the primary evaluation of obese children.

## Ethics

Ethics Committee Approval: The present study was approved by the local ethics committee, Informed Consent: It was taken.

Peer-review: External peer-reviewed.

## Figures and Tables

**Table 1:**
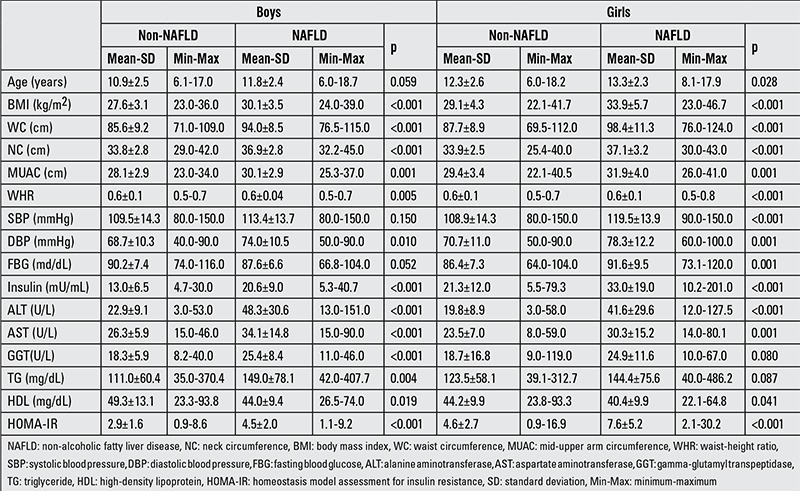
Comparison of subjects with and without non-alcoholic fatty liver disease in terms of metabolic and anthropometric parameters in boys and girls

**Table 2:**
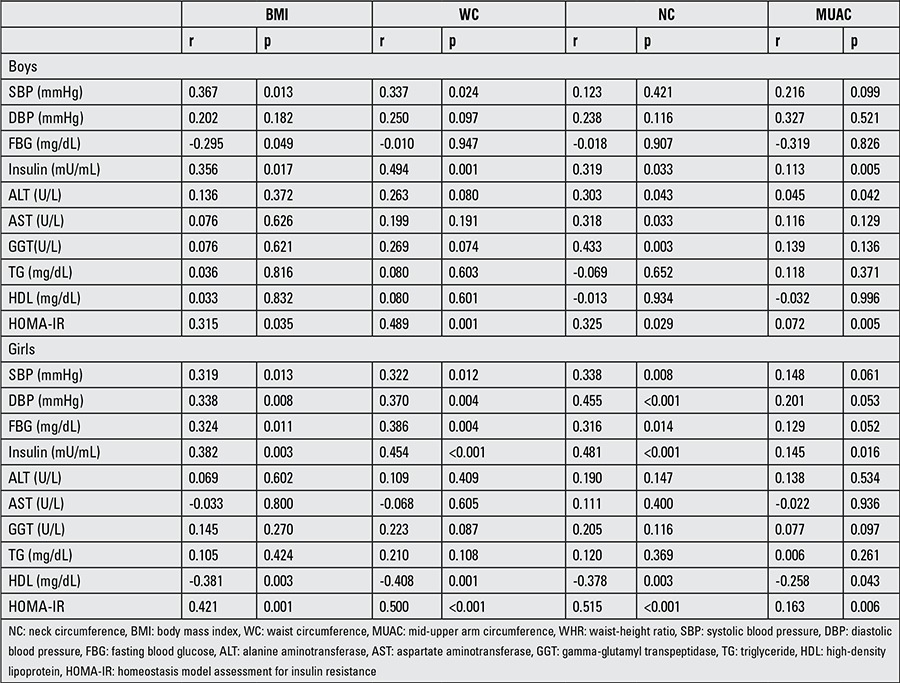
Correlations between anthropometric and metabolic parameters after adjusting for age and pubertal stage

**Table 3:**
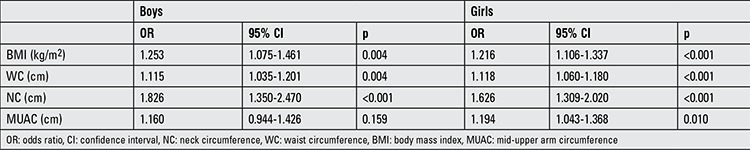
Univariate logistic regression analysis between having non-alcoholic fatty liver disease and anthropometric parameters in boys and girls

**Table 4:**
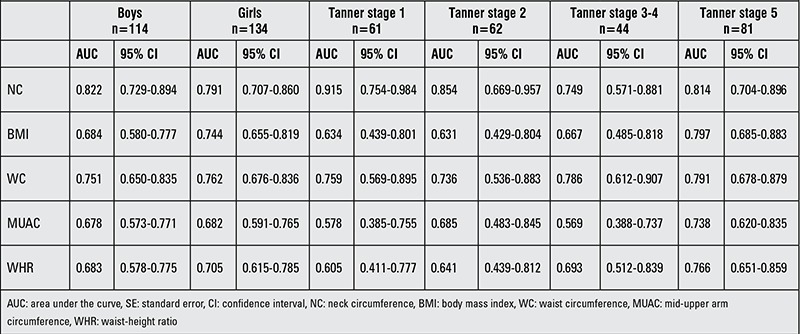
Comparison of anthropometric parameters by receiver operating curves analysis in defining non-alcoholic fatty liver disease according to gender and pubertal stage

**Table 5:**
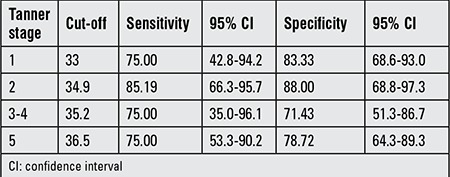
Neck circumference cut-off values for determining non-alcoholic fatty liver disease according to pubertal stages
